# Supplementary material for: The interactome of intact mitochondria by cross-linking mass spectrometry provides evidence for coexisting respiratory supercomplexes
Source: Mol Cell Proteomics. 2017 Dec 8;17(2):216–32. doi: 10.1074/mcp.RA117.000470 (PMC5795388; doi:10.1074/mcp.RA117.000470)
Supplement: Supplemental Data [file supp_RA117.000470_133922_0_supp_23978_fzffwf.zip › spectra_annotation/mito_DR_spectra_annotation/294-1-12-1-11-1.pdf]

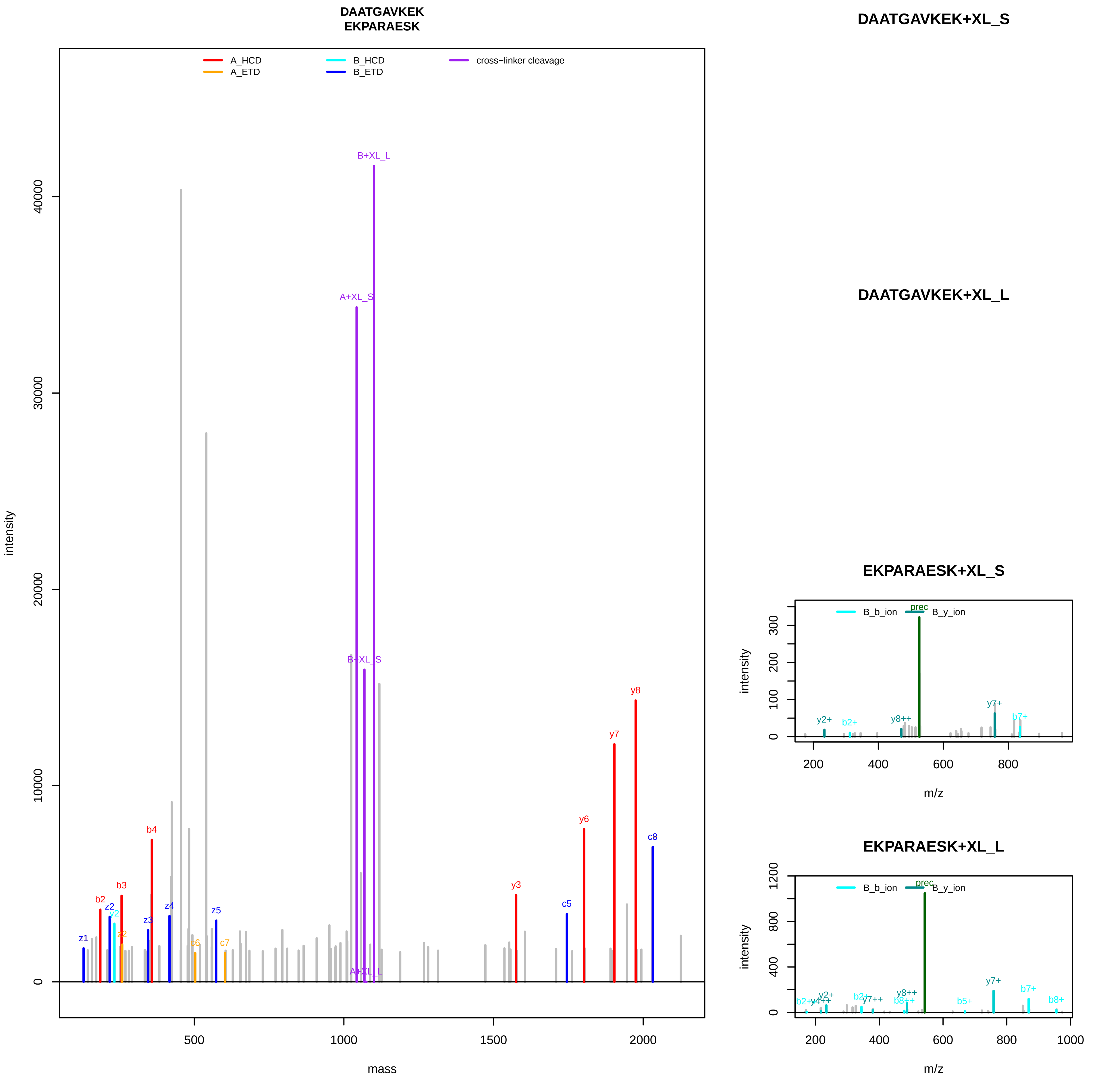
DAATGAVKEK
EKPARAESK
A_HCD
A_ETD
B_HCD
B_ETD
cross−linker cleavage
intensity
mass
B+XL_L
A+XL_S
B+XL_S
A+XL_L
z1
b2
z2
y2
b3
z2
z3
b4
z4
z5
c6
c7
y3
c5
y6
y7
y8
c8
DAATGAVKEK+XL_S
DAATGAVKEK+XL_L
EKPARAESK+XL_S
EKPARAESK+XL_L
B_b_ion
B_y_ion
prec
m/z
y2+
b2+
y8++
y7+
b7+
b2++
y4++
b8++
b5+
b8+
